# The role of neuronal excitation and inhibition in the pre-Bötzinger complex on the cough reflex in the cat

**DOI:** 10.1152/jn.00108.2021

**Published:** 2021-12-08

**Authors:** Tabitha Y. Shen, Ivan Poliacek, Melanie J. Rose, M. Nicholas Musselwhite, Zuzana Kotmanova, Lukas Martvon, Teresa Pitts, Paul W. Davenport, Donald C. Bolser

**Affiliations:** ^1^Department of Physiological Sciences, College of Veterinary Medicine, University of Florida, Gainesville, Florida; ^2^Jessenius Faculty of Medicine in Martin, Institute of Medical Biophysics, Comenius University in Bratislava, Martin, Slovak Republic; ^3^Department of Neurological Surgery, Kentucky Spinal Cord Injury Research Center, University of Louisville, Louisville, Kentucky

**Keywords:** cough, DLH, gabazine, kynurenic acid, pre-Bötzinger complex

## Abstract

Brainstem respiratory neuronal network significantly contributes to cough motor pattern generation. Neuronal populations in the pre-Bötzinger complex (PreBötC) represent a substantial component for respiratory rhythmogenesis. We studied the role of PreBötC neuronal excitation and inhibition on mechanically induced tracheobronchial cough in 15 spontaneously breathing, pentobarbital anesthetized adult cats (35 mg/kg, iv initially). Neuronal excitation by unilateral microinjection of glutamate analog d,l-homocysteic acid resulted in mild reduction of cough abdominal electromyogram (EMG) amplitudes and very limited temporal changes of cough compared with effects on breathing (very high respiratory rate, high amplitude inspiratory bursts with a short inspiratory phase, and tonic inspiratory motor component). Mean arterial blood pressure temporarily decreased. Blocking glutamate-related neuronal excitation by bilateral microinjections of nonspecific glutamate receptor antagonist kynurenic acid reduced cough inspiratory and expiratory EMG amplitude and shortened most cough temporal characteristics similarly to breathing temporal characteristics. Respiratory rate decreased and blood pressure temporarily increased. Limiting active neuronal inhibition by unilateral and bilateral microinjections of GABA_A_ receptor antagonist gabazine resulted in lower cough number, reduced expiratory cough efforts, and prolongation of cough temporal features and breathing phases (with lower respiratory rate). The PreBötC is important for cough motor pattern generation. Excitatory glutamatergic neurotransmission in the PreBötC is involved in control of cough intensity and patterning. GABA_A_ receptor-related inhibition in the PreBötC strongly affects breathing and coughing phase durations in the same manner, as well as cough expiratory efforts. In conclusion, differences in effects on cough and breathing are consistent with separate control of these behaviors.

**NEW & NOTEWORTHY** This study is the first to explore the role of the inspiratory rhythm and pattern generator, the pre-Bötzinger complex (PreBötC), in cough motor pattern formation. In the PreBötC, excitatory glutamatergic neurotransmission affects cough intensity and patterning but not rhythm, and GABA_A_ receptor-related inhibition affects coughing and breathing phase durations similarly to each other. Our data show that the PreBötC is important for cough motor pattern generation, but cough rhythmogenesis appears to be controlled elsewhere.

## Graphical abstract

**Figure:**
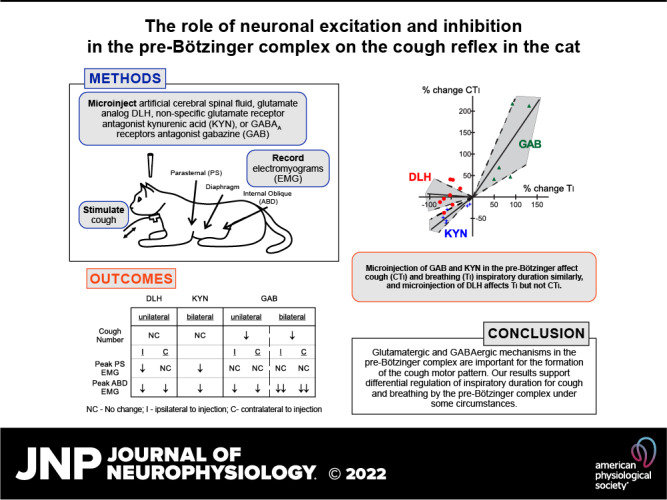

## INTRODUCTION

Regulation of respiratory functions represents a complex interaction of chemodetection, generation of the respiratory rhythm, adjustments to various conditions and also other competing behaviors, and effective control of spatiotemporal characteristics of the respiratory pattern. The pre-Bötzinger complex (PreBötC), located in the rostral ventral respiratory column, contains respiratory neurons of variable bursting characteristics and interconnections that form the assembly generating inspiratory rhythm and pattern (1–5). As such, the area represents a crucial kernel for the breathing rhythm and pattern generation and likely for many other respiratory adaptive functions (6–8).

Data from neuronal recording experiments and modeling approaches strongly support the view that respiratory neurons in respiratory-related brainstem locations, including the PreBötC, form a common breathing/cough central pattern generator (9–16). The term “common” reflects the fact that a significant portion of the respiratory neuronal network is involved in the production of motor patterns for other behaviors, particularly cough. The results of previous studies using neuroactive substances in respiratory-related areas are consistent with this hypothesis. For example, excitation of neurons in the caudal ventral respiratory column with d,l-homocysteic acid (DLH), a nonspecific excitatory amino acid receptor agonist, resulted in an increased tonic component of expiratory motor activity in the expiratory transversus abdominis muscle during breathing and a significant reduction in the number of coughs and their expiratory motor component (17). Furthermore, alterations in the activity of neurons in the Bötzinger complex (BötC) perturbed coughing (11, 18). Moreover, several neurotransmitter mechanisms are involved in cough modulation from distinct respiratory brainstem areas (5, 13, 16–24). A frequent observation during studies that have used microinjection of neuroactive substances into areas that modulate the breathing pattern was differential effects on the breathing and cough motor patterns, which is explained by presumptive differing functions of respiratory neuronal assemblies when generating eupnea and cough. This process has been termed “reconfiguration.” The process of reconfiguration to generate the cough motor pattern when cough-related afferent inputs activate nucleus tractus solitarius (NTS), second-order neurons can include the recruitment of neurons that are silent during breathing but active during coughing (behavior-specific), as well as changes in the phase and/or magnitude of major activation of spontaneously active of neurons in the network (retasking) (9, 10, 25, 26).

In this study, we attempted to induce changes in breathing and coughing in an anesthetized cat animal model via alterations in glutamate and GABA-related neurotransmission in the PreBötC. We hypothesized that the PreBötC will significantly alter spatiotemporal motor pattern consistent with differential regulation of breathing and coughing behaviors.

## METHODS

### Animals

Experiments were performed on 15 cats (5.37 ± 0.18 kg, 1 female and 14 males). These cats were purchased from Marshall BioResources (North Rose, NY) and pair-housed in the University of Florida Animal Care Services on a 12-h light/12-h dark cycle with food and water ad libitum.

### Ethics Statement

All procedures were performed in accordance with the *Guide for the Care and Use of Laboratory Animals* and approved by the University of Florida Institutional Animal Care and Use Committee. Great care was taken to minimize animal suffering and numbers.

### Surgical Procedure

The animals were induced with 3%–5% sevoflurane (Patterson Veterinary, Greeley, CO) and weaned onto sodium pentobarbital (25 mg/kg iv; Akorn Pharmaceuticals, Lake Forest, IL). Anesthetic depth was assessed every 15 min. When necessary (depending on presence of corneal and forelimb pullback reflex, jaw tone, respiratory rate, and blood pressure), supplemental anesthesia was administered (1–3 mg/kg iv). Atropine sulfate (0.054 mg/kg iv; Med-Pharmex, Pomona, CA) was given at the beginning of the experiment to reduce airway secretions. The trachea, femoral artery, and vein were cannulated. The animals were allowed to breathe spontaneously. If necessary, oxygen was added in the inhaled air (∼40% oxygen, balance nitrogen). Arterial blood pressure (BP; 1902 isolated preamplifier, CED, Oxford, UK), respiratory rate, end-tidal CO_2_ (ETCO2; GEMINI O2 & CO2 Monitor, CWE Inc., Ardmore, PA), and body temperature were continuously monitored, and temperature was maintained at 38.0 ± 0.5°C by a heating pad (TC-1000 Cat Temperature Control System, CWE Inc., Ardmore, PA). Hourly samples of arterial blood were removed for blood gas and pH analysis (Element POC, Heska, Loveland, CO). The cats were euthanized by an overdose of pentobarbital (iv) followed by 3 mL of a saturated potassium chloride solution (iv) (Thermo Fisher Scientific, Waltham, MA).

### EMG Recordings

Electromyograms (EMG) were recorded with bipolar, insulated fine wire hook electrodes bilaterally from the internal oblique (abdominal, ABD) muscle and inspiratory parasternal (PS) muscles and sternal diaphragm (DIA). The PS electrodes were placed at T3 proximal to the sternum after exposing the surface of the muscle. DIA electrodes were inserted just under the xiphoid process through intact abdominal muscles.

### Microinjection Procedures

Animals were placed prone in a stereotaxic frame and the dorsal surface of medulla was exposed by an occipital craniotomy. The head was ventroflexed by 10°. The surface of the brainstem was covered by mineral oil soaked felt between microinjections. Carbon electrode multibarrel micropipette composites (Carbostar-3 or Carbostar-4, Kation Scientific, Minneapolis, MN) were used for pressure injections of the solutions. The injected volume was monitored by observation of movement of the meniscus in the micropipette with a microscope. Accounting for the 10° flexure, the micropipette was positioned under stereotaxic control such that the tip was in the region of the PreBötC (∼3.5 mm rostral to the obex, 3.7 mm lateral from the midline, and 4.5 mm under the dorsal medullary surface; all coordinates reported are calculated such that they are stereotaxic coordinates). Appropriate location was confirmed by the presence of inspiratory-modulated multiunit neuronal activity, since neurons in the BötC are almost purely expiratory neurons, and the PreBötC is a mixture of both inspiratory and expiratory neurons (27–31). In addition, the positioning of the micropipette tip in the PreBötC was ascertained by the respiratory response to microinjection of DLH (Sigma-Aldrich, St. Louis, MO) or gabazine (GAB; Tocris, Minneapolis, MN) and by the presence of a fluorescence marker [Fluospheres carboxylate-modified, 0.04 μm red (580/605) or yellow-green (505/515), Thermo Fisher Scientific, Waltham, MA] in histological sections at the correct anatomical location. The fluorescent beads were suspended in the injectate of DLH, kynurenic acid (KYN; Sigma-Aldrich, St. Louis, MO), GAB, or artificial cerebrospinal fluid (aCSF; Harvard Apparatus, Holliston, MA). In 8 of the 15 cats, DLH (5 mM solution in aCSF, 25–50 nL) (32, 33) was microinjected unilaterally (positioning: 3.0–3.8 mm rostral to the obex, 3.5–3.8 mm lateral from the midline, 4.2–5.0 mm below the dorsal medullary surface). Physiological evidence that the electrode tip was in the PreBötC region included tonic inspiratory muscle EMG activity followed and/or overlapped by a high breathing rate (fB) (32) induced by microinjection of DLH (see results) within the first 1–2 min post injection. In six of the eight cats previously microinjected with DLH, KYN (50 mM solution in aCSF, 34–50 nL) (23) was later microinjected bilaterally in the same location as the DLH microinjections. The other two of the eight cats microinjected with DLH were subjected to unilateral microinjections of aCSF following unilateral microinjections of DLH. Twenty to sixty minutes elapsed between DLH and subsequent microinjections. In a separate group of 5 of the 15 cats, GAB solutions (0.1 mM in aCSF, 25–50 nL) (34, 35) were microinjected bilaterally. One of these five animals was previously tested with bilateral microinjection of aCSF and two of these five animals were microinjected with bilateral aCSF (20–50 nL) 120–200 min after the recovery from GAB microinjections. An additional 2 of the 15 cats underwent solely bilateral aCSF control microinjections. Altogether, five animals were microinjected bilaterally with aCSF, and two were microinjected unilaterally with aCSF.

### Histology

**Figure 1.**
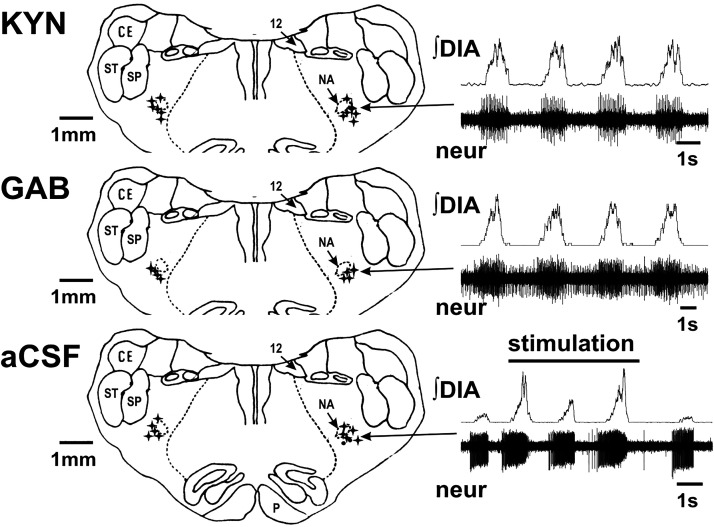
Schematic reconstruction of microinjection sites in the pre-Bötzinger complex area around nucleus ambiguus (NA). The transverse section on the figure is ∼3.5 mm rostral to the obex. The injections were from 3.0 to 4.2 mm rostral to the obex, 0.3 to 1.0 mm caudal from the caudal-most edge of retrofacial nucleus, and were projected to this schematic section. Using histological methods, we located all 12 microinjections of kynurenic acid (KYN, the first panel), 8 out of 8 microinjections of gabazine (GAB) that contained the fluorescent marker (GAB, the second panel; GAB solution had no marker in 1 cat) and all 12 control artificial cerebrospinal fluid microinjections (CSF, the third panel). Ten of these microinjections were delivered bilaterally (stars) and an additional 2 were solely unilateral (dots below NA on the *right* side). *Inset* recordings (right-hand side) represent examples of integrated diaphragm (∫DIA) and extracellular neuronal activity (neur) recorded at the microinjection site. Arrows show the location of the microelectrode recording site. The response of a single putative preinspiratory neuron during coughing after microinjection of artificial cerebrospinal fluid (aCSF) is shown in the *bottom* panel. The occurrence of coughs are shown by the larger inspiratory bursts (abdominal bursts not shown). CE, external cuneate ncl.; P, pyramidal tract; SP, alaminar spinal trigeminal ncl.; ST, spinal trigeminal tract; 12, hypoglossal ncl.

After the experiment, the medulla was removed and the tissue was fixed in 4% paraformaldehyde (Fisher Scientific, Waltham, MA) followed by 30% sucrose (Fisher Scientific, Waltham, MA) solution. The frozen medulla was then cut into transverse slices (thickness 50 or 100 µm) by a freezing microtome (Microm HM505E, Microm GmbH, Walldorf, Germany). Sections were examined under light and UV microscopy (All-in-One Fluorescence Microscope BZ-X710, Keyence, Itasca, IL) for detection and localization of injection sites in PreBötC (32, 36). Different colors of fluorescent dye were used if both aCSF and GAB were microinjected in the same animal (in 1 case the GAB solution did not contain fluorescent beads). Reconstruction of injected sites relative to the nucleus ambiguus is summarized in Fig. 1. Microinjections of KYN were found 3.1–3.6 mm rostral to the obex, 3.4–4.2 mm lateral to the midline, and 3.9–5.6 mm below the dorsal medullary surface. The GAB microinjection sites were at coordinates 3.0–4.2/3.5–3.9/4.0–4.9 and those for aCSF into the range 3.1–3.5/3.6–4.0/4.0–5.0.

### Tracheobronchial Cough Stimulation

Tracheobronchial cough was elicited by mechanical stimulation of the intrathoracic airways with a thin polyethylene catheter. This catheter was inserted into the trachea for 10 s [moved back and forth (1 Hz) and rotated (10 Hz) to elicit repetitive coughing]. In two animals, 20-s stimulation trials were conducted. Cough was defined by a large burst of inspiratory-related PS and diaphragm EMG activity immediately followed by a burst of expiratory ABD EMG activity. These criteria separated cough from other airway defensive behaviors such as expiration reflex, augmented breath, aspiration reflex, and swallow (18, 26).

### Protocol

First, 15–25 consecutive cough stimulation trials, separated by ∼1 min, were conducted to establish a stable cough baseline. Then, 2–5 control cough trials were completed, followed by 2–5 trials in each postmicroinjection testing periods: 0–2 min for DLH, 5–35 min for KYN, within 8 min after unilateral, and within 30 min after bilateral microinjection of GAB. Then, two to five cough stimulation trials were conducted every hour until recovery was observed. Control coughs for aCSF microinjection were analyzed before, right after unilateral injection, and then 5 min after the second of bilateral microinjections.

### Data Analysis

All EMGs were amplified and filtered (300–5,000 Hz; Grass P511, Astro-Med, West Warwick, RI). Offline, the data were rectified and smoothed (moving average time constant 200 ms) (Spike2 Version 9, Cambridge Electronic Design Limited, Cambridge, UK). The following cough data were analyzed: the number of coughs (CN) in response to mechanical stimulation of the trachea (cough number = average number of coughs per 10 s stimulation per trial), amplitude of PS (CPkps), DIA (CPkdia), and ABD EMG (CPkabd) moving averages, amplitude of esophageal pressure (EPi, inspiratory trough activity; EPe, expiratory peak activity) during appropriate phases, the inspiratory (CTi, augmenting/elevating part of PS burst) and expiratory (CTe, from the peak of PS/DIA discharge to the end of cough cycle) phase durations, the active portion of cough expiration (CTe1, from the peak of PS activity to the end of cough-related ABD discharge), expiratory “passive” period (CTe2, from the end of cough ABD discharge to the end of the cough cycle), total cough cycle time (CTtot), and duration of cough-related motor activity (CTma).

**Figure 2.**
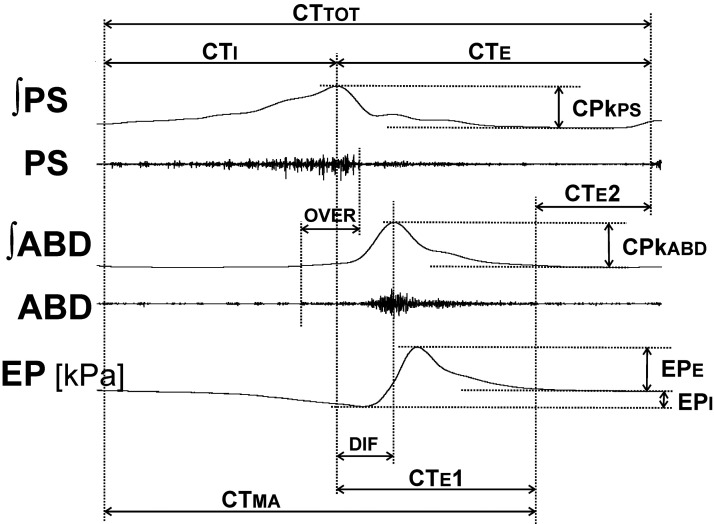
Illustration of spatiotemporal cough measurements. ABD, abdominal muscles; CPkabd, abdominal muscle EMG, amplitude of ʃABD (within arrows); CPkps, parasternal muscle EMG; CTabd, durations of abdominal muscle cough-related discharge; CTE, duration of the cough expiratory phase; CTE1, duration of active cough expiratory phase; CTE2, duration of the quiescent period of cough expiration; CTI, duration of cough inspiratory phase; CTma, duration of cough-related EMG activity; CTtot, total cough cycle duration; dif, time interval between the peaks of parasternal/diaphragm and abdominal EMG activity; EMG, electromyogram; EP, esophageal pressure; EPe, EPi, expiratory and inspiratory esophageal pressure amplitudes, respectively; ʃABD, integrated moving average of the abdominal muscle EMG; ʃPS, integrated moving average of the parasternal muscle EMG; over, duration of parasternal/diaphragm and abdominal EMG coactivation; PS, parasternal muscles. Note that diaphragm EMG (which is practically identical to PS activity) is not shown.

In addition, other features of the cough motor pattern were analyzed: the overlap of cough-related PS/DIA and ABD discharge (over), the time between peaks of PS/DIA and ABD activity (dif). The variables for cough analysis are illustrated in Fig. 2. The data were compared in control preinjection and postinjection periods.

Magnitudes of the moving averages during coughing were normalized relative to the mean intensities of control preinjection coughs. Percent change in CN compared with control was calculated (CN%). All cough parameters were the result of averaging all coughs induced during all trials within each testing segment of the protocol. Each of five protocols (unilateral DLH, bilateral KYN, unilateral followed by bilateral GAB, unilateral aCSF, and unilateral followed by bilateral aCSF microinjections) was analyzed independently.

Analyzed cardiorespiratory data included fB; DIA EMG amplitude (Pkdia) during breathing; inspiratory (onset to peak of PS/DIA EMG activity, Ti), postinspiratory (peak to termination of PS/DIA EMG activity, Te1), and expiratory (peak of PS/DIA EMG activity to onset of PS/DIA EMG activity of the next breath) phase durations (Te, Te2, where Te = Te1+ Te2); mean arterial blood pressure (MAP); heart rate; and ETCO2. Monitored cardiorespiratory parameters were measured during three to five consecutive respiratory cycles in the control period before the first microinjection and in the postinjection periods. The timepoints of postmicroinjection cardiorespiratory analysis differed. These values were collected within a minute for DLH microinjections, within 5–20 min after the second of bilateral microinjections for KYN and within 5 min after the first and then 5–20 min after the second delivery of GAB. We measured cardiorespiratory variables at or near the maximum of their changes induced by microinjections.

### Statistics

Results are expressed as means ± SE. In the graphs, the median, 95% confidence interval, and minimum and the maximum of the data are shown. Statistical analysis was performed using InStat (GraphPad, San Diego, CA). Normality of the data was tested using the Kolmogorov–Smirnov normality test. For statistical analysis of more than two groups, we used a repeated-measures ANOVA (RM-ANOVA) or an ordinary ANOVA with a Student–Newman–Keuls posttest for normally distributed data. If data did not pass the normality test, then group data were tested using the Kruskal–Wallis ANOVA with a Dunn’s posttest. For statistical analysis comparing two groups, normally distributed data were analyzed using an unpaired *t* test. The differences of variables were considered significant if *P* < 0.05.

## RESULTS

**Figure 3.**
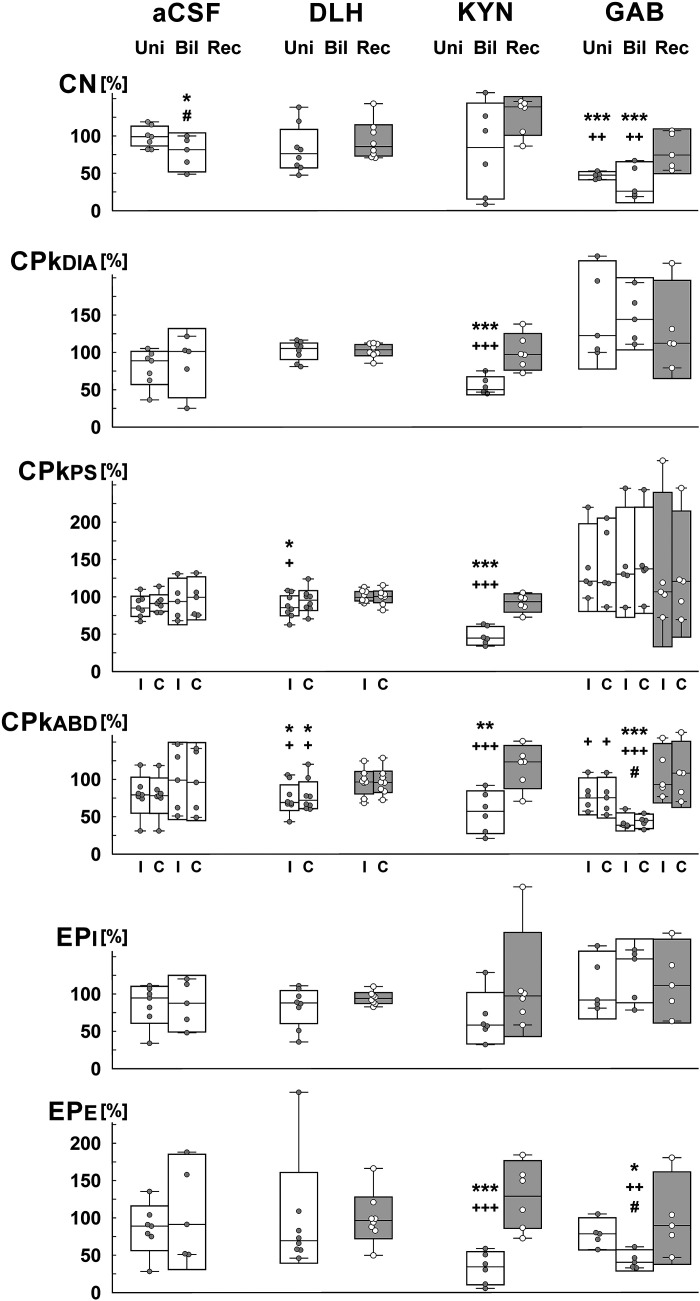
Relative measures of spatial (intensity) cough characteristics after microinjections of d,l-homocysteic acid (DLH), kynurenic acid (KYN), and gabazine (GAB) in the pre-Bötzinger complex area. Boxes represent the median and 95% confidence interval, short lines attached to the lower and upper interval limit are minimum and maximum value in the data set, respectively. Sample size: 8 for DLH, 6 for KYN, 5 for GAB, 7 for aCSF (5 bilateral, 7 unilateral); repeated-measures ANOVA was used for statistical analysis, but ordinary ANOVA for contralateral ABD with DLH microinjection, for ABD and EP_E_ with KYN microinjections; *,**,***P < 0.05, <0.01, <0.001 compared with preinjections, respectively; +,++,+++*P* < 0.05, <0.01, <0.001 compared with the recovery, respectively; #*P* < 0.05 compared with unilateral delivery data. aCSF, artificial cerebrospinal fluid; Bil, after bilateral microinjection; C, contralateral; CN, number of coughs per 10 s stimulus (per 1 trial); DIA, PS, ABD, normalized amplitudes of the diaphragm, parasternal muscles, and abdominal muscles electromyogram (EMG) moving averages; EPi, EPe, inspiratory and expiratory esophageal pressure amplitudes, respectively; I, ipsilateral; Rec, in the recovery period; Uni, after unilateral microinjection.

**Figure 4.**
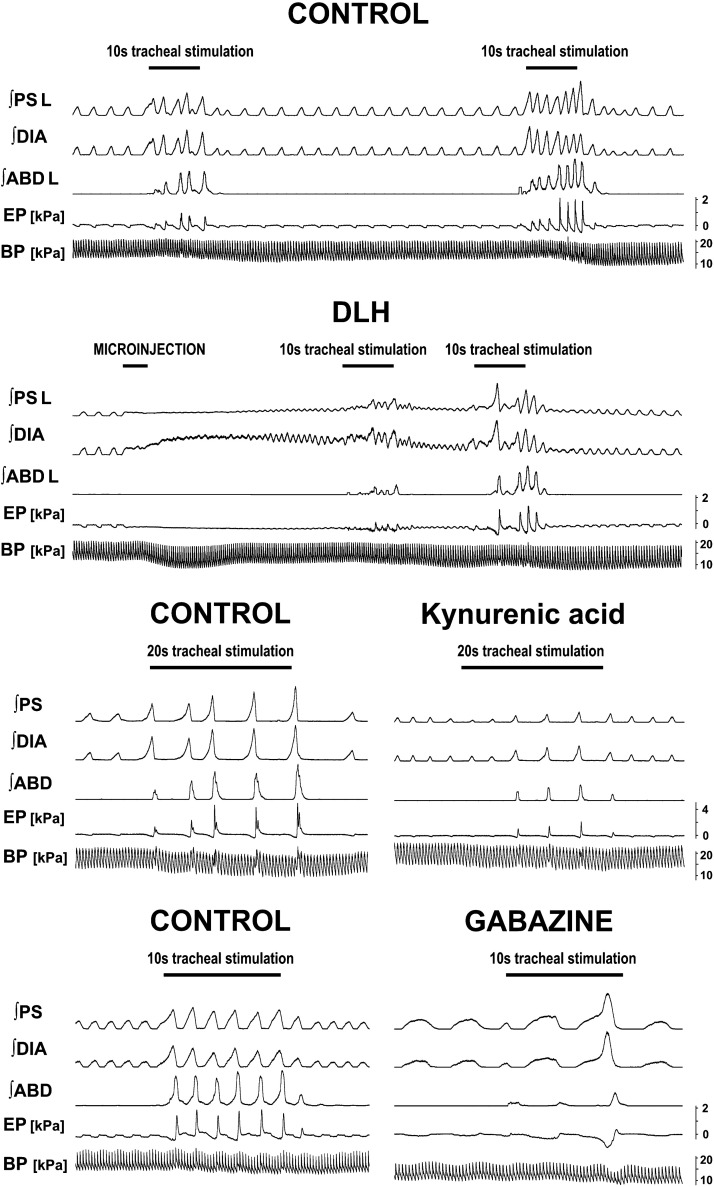
Examples of cough (and respiratory) changes induced by microinjections in the pre-Bötzinger complex area. The first panel represents 2 control pre-d,l-homocysteic acid (DLH) cough trials. The second panel shows 2 cough trials during respiratory changes induced by microinjection of DLH. The third panel displays a typical cough trial in control and 6 min after bilateral microinjections of kynurenic acid (KYN). The forth panel demonstrates changes induced by bilateral microinjections of gabazine (GAB) (postinjection trial at 3 min after the bilateral delivery). ABD, abdominal muscles; BP, arterial blood pressure; DIA, diaphragm; EP, esophageal pressure; ∫, integrated EMG; L, the left side (ipsilateral to DLH microinjection is shown); PS, parasternal muscles.

Microinjections of neuroactive compounds in the PreBötC region elicited significant changes of coughing and breathing (Figs. 3 and 4 and Table 1). The volumes of our microinjections are very unlikely to have directly modified the activity of neurons at a distance more than 0.5 mm away from the micropipette tip (17, 23, 37–39).

**Table 1.** Temporal cough parameters

	Control	Unilateral	Bilateral	Recovery	*P* Value
CTi, s					
DLH	1.00 ± 0.07	1.06 ± 0.11		0.97 ± 0.09	NS
KYN	1.13 ± 0.13		0.66 ± 0.06**‡	0.90 ± 0.04*	<0.01
GAB	0.99 ± 0.09	1.80 ± 0.17*	2.12 ± 0.39**‡	1.28 ± 0.16	<0.01
CTe, s					
DLH	1.36 ± 0.21	1.62 ± 0.43		1.59 ± 0.24	NS
KYN	1.81 ± 0.27		1.41 ± 0.30*	1.34 ± 0.14*	<0.05
GAB	2.00 ± 0.66	4.35 ± 1.59*‡	5.61 ± 1.38**‡‡	2.18 ± 0.69	<0.01
CTe1, s					
DLH	0.80 ± 0.09	0.68 ± 0.09*‡		0.81 ± 0.09	<0.05
KYN	0.96 ± 0.11		0.83 ± 0.04	0.85 ± 0.03	NS
GAB	0.88 ± 0.05	1.46 ± 0.23	1.83 ± 0.27*‡	0.97 ± 0.06	<0.05
CTe2, s					
DLH	0.57 ± 0.14	0.94 ± 0.37		0.77 ± 0.17	NS
KYN	0.86 ± 0.19		0.58 ± 0.27	0.50 ± 0.16*	<0.05
GAB	1.11 ± 0.70	2.89 ± 1.73	3.79 ± 1.50	1.21 ± 0.67	=0.05
CTtot, s					
DLH	2.36 ± 0.26	2.68 ± 0.52		2.55 ± 0.29	NS
KYN	2.95 ± 0.38		2.07 ± 0.34**	2.25 ± 0.16**	<0.01
GAB	2.99 ± 0.74	6.15 ± 1.65*‡	7.73 ± 1.41***‡‡	3.45 ± 0.80	<0.001
CTma, s					
DLH	1.80 ± 0.15	1.74 ± 0.19		1.78 ± 0.16	NS
KYN	2.09 ± 0.23		1.48 ± 0.08*	1.75 ± 0.04	<0.05
GAB	1.88 ± 0.08	3.26 ± 0.26*	3.94 ± 0.65**‡	2.24 ± 0.16	<0.01
dif, s					
DLH	0.23 ± 0.02	0.27 ± 0.01		0.25 ± 0.01	NS
KYN	0.28 ± 0.02		0.26 ± 0.02	0.25 ± 0.01	NS
GAB	0.27 ± 0.03	0.40 ± 0.04†††	0.64 ± 0.05***‡‡‡	0.28 ± 0.03	<0.001
over, s					
DLH	0.43 ± 0.06	0.34 ± 0.04		0.45 ± 0.05	NS
KYN	0.57 ± 0.09		0.31 ± 0.07**‡	0.47 ± 0.10	<0.05
GAB	0.51 ± 0.10	0.87 ± 0.22	0.80 ± 0.34	0.57 ± 0.16	NS

Values are means ± SE. Bilateral, pooled data for 1–8 and 20–30 min for gabazine (GAB), pooled data for 5–15 and 20–35 min post last microinjection for kynurenic acid (KYN); control, preinjection control; recovery, 5–35 min for DLH, >120 min for KYN and GAB after the last microinjection; unilateral, within 120 s postmicroinjection for d,l-homocysteic acid (DLH), 1–8 min for GAB. CTe, duration of the cough expiratory phase; CTe1, duration of active cough expiratory phase; CTe2, quiescent period of cough expiration; CTi, duration of cough inspiratory phase; CTtot, total cough cycle duration; dif, time interval between the peaks of parasternal/diaphragm and abdominal EMG activity; over, duration of parasternal/diaphragm and abdominal EMG activities overlap. Sample size: 8 for DLH, 6 for KYN, 5 for GAB; *P* values in the table are that of the repeated-measures ANOVA, *P* values from the Student–Newman–Keuls posttest; *, **, ***P < 0.05, <0.01, <0.001 compared with preinjection control, respectively; †††*P* < 0.001 compared with bilateral data; ‡, ‡‡, ‡‡‡*P* < 0.05, <0.01, <0.001 compared with recovery, respectively; not significant (NS): *P* > 0.05.

### Effects on Cough

#### DLH.

Unilateral microinjection of DLH into the PreBötC (5 mM DLH, 11 microinjections unilaterally, 44 ± 3 nL per injection, 0.30 ± 0.05 nmol per injection, 8 cats) resulted in a reduced CPkabd [to 75 ± 7% ipsilateral (RM-ANOVA) and to 79 ± 8% contralateral (ordinary ANOVA) amplitudes of EMG moving averages relative to control values, recovery 95 ± 6% and 97 ± 6%, respectively] and ipsilateral CPkps (to 88 ± 6% relative to control values, recovery 101 ± 3%, RM-ANOVA) during cough (Figs. 3 and 4) along with shortened CTe1 (Table 1). CN during the control period was 4.88 ± 0.66.

#### Kynurenic acid.

The effect of microinjection of KYN (50 mM, bilaterally, 44 ± 3 nL for single injections, 4.34 ± 0.13 nmol for bilateral delivery, 6 cats) included: reduced inspiratory motor drive (EMG amplitudes relative to control CPkdia 55 ± 5% and CPkps 48 ± 5%, recovery 101 ± 10% and 92 ± 5%, respectively, RM-ANOVA, Figs. 3 and 4), reduced expiratory efforts (CPkabd and EPe at 56 ± 11% and 33 ± 9%, relative to control, respectively, recovery 117 ± 11% and 131 ± 18%, respectively, ordinary ANOVA, Figs. 3 and 4), and shortened CTi, CTe, CTtot, CTma, and over (RM-ANOVA, Fig. 4 and Table 1). CN during the control period was 4.45 ± 0.55.

#### Gabazine.

The volumes and quantities of GAB (0.1 mM) microinjections were 42 ± 3 nL, 4.2 ± 0.3 for unilateral injections and 40 ± 2 nL, 7.9 ± 3 pmol per injection for bilateral injections. GAB markedly reduced CN (during control: 5.09 ± 0.57; CN%: unilateral-bilateral-recovery: 47 ± 2%-38 ± 10%-80 ± 11%, RM-ANOVA, Figs. 3 and 4), cough frequency (unilateral-bilateral-recovery: 48 ± 4%-42 ± 10%-90 ± 11%, *P* < 0.001 both microinjection conditions compared with control and with recovery, RM-ANOVA). Expiratory cough efforts were also reduced by unilateral and bilateral microinjections of GAB (CPkabd: ipsilateral side—unilateral 77 ± 9%, bilateral 43 ± 4%, recovery 108 ± 14%; contralateral side—unilateral 76 ± 10%, bilateral 44 ± 4%, recovery 107 ± 16%; CPkep: unilateral 79 ± 8%, bilateral 43 ± 5%, recovery 100 ± 22%, RM-ANOVA, Figs. 3 and 4). Temporal changes in the cough motor pattern included statistically significant prolongation of CTi, CTe, CTe1, CTtot, CTma, and dif (RM-ANOVA, Fig. 4 and Table 1).

#### Artificial cerebrospinal fluid.

Control aCSF microinjections (unilateral 8 microinjections in 7 cats, 36 ± 3 nL per injection) had no significant effect on cough (Fig. 3). Similarly, bilateral microinjections of aCSF (5 cats, 82 ± 6 nL bilateral delivery) induced no significant alterations in cough responses, with the exception of reduced CN after bilateral microinjection (CN during control: 3.95 ± 0.55; CN%: 2.96 ± 0.75–78 ± 9%, *P* < 0.05, RM-ANOVA, compared with control: 3.75 ± 0.75–100%). The CN after bilateral microinjections of aCSF was significantly different than this metric of cough after preceding unilateral aCSF microinjections (3.63 ± 0.70–99 ± 6%, RM-ANOVA), which were not significantly different from control. This value (78 ± 9% of control) was significantly greater than the effect of bilateral microinjection of GAB on CN (38 ± 10% of control, *P* < 0,05, unpaired *t* test), indicating a drug effect relative to vehicle.

### Cardiorespiratory Changes

Microinjections in the PreBötC induced significant cardiorespiratory changes.

#### DLH.

Unilateral DLH delivery induced immediate and short-lasting (up to 2 min) tonic excitation of inspiratory motor output (DIA and PS EMG discharge) followed by and overlapping with high-frequency respiratory DIA and PS bursts (Fig. 4). The duration of apneusis was 15.5 ± 3.0 s and the tonic component of inspiratory motor output (that includes the period of apneusis) lasted 58.5 ± 9.3 s. The rapid fB that occurred during the tonic inspiratory activity reached 252 ± 25% of control (*P* < 0.001 to control and recovery 102 ± 9%, RM-ANOVA). Ti consistently shortened to 0.45 ± 0.05 s from control 0.93 ± 0.05 s (recovery 1.01 ± 0.08 s, *P* < 0.001 for both, RM-ANOVA) and Te decreased to 0.84 ± 0.11 s from 2.17 ± 0.19 s (recovery 2.11 ± 0.21 s, *P* < 0.001 for both, RM-ANOVA) and Te2 was reduced to 0.44 ± 0.10 s from 1.96 ± 0.20 s (recovery 1.91 ± 0.22 s, *P* < 0.001 for both) (Fig. 4). However, Te1 was prolonged to 0.40 ± 0.04 s from 0.22 ± 0.01 s (recovery 0.20 ± 0.02 s, *P* < 0.001 for both, RM-ANOVA). The amplitude of inspiratory EMG tonic activity induced by microinjections of DLH was mostly lower than that during control breathing, but the amplitude of the fast inspiratory bursts during the tonic inspiratory activity reached 136 ± 13% of control (*P* < 0.01 to control and recovery 97 ± 3%, RM-ANOVA, Fig. 4). ETCO2 decreased during the period of rapid breathing to 3.5 ± 0.4 from 4.2 ± 0.2 kPa (recovery 4.4 ± 0.1 kPa, *P* < 0.05 for both, RM-ANOVA). MAP also decreased (Fig. 4) to 12.4 ± 0.9 from 16.8 ± 0.7 kPa (recovery 16.5 ± 0.7 kPa, *P* < 0.01 to both, RM-ANOVA), but there were no significant changes in heart rate.

#### Kynurenic acid.

Bilateral microinjections of KYN resulted in increased fB to 187 ± 25% of control (*P* < 0.05, RM-ANOVA) that occurred within a 5-min interval following the injection (Fig. 4), lasted several hours, and very slowly started to recover (recovery 173 ± 27%, *P* < 0.05 vs. control, RM-ANOVA). Ti consistently shortened (Fig. 4) from 1.09 ± 0.08 s to 0.62 ± 0.15 s (*P* < 0.05) and did not recover in the time span for cough to return close to control values (recovery 0.74 ± 0.13 s, *P* < 0.05 to control); Te (Fig. 4) decreased from 2.14 ± 0.32 s to 1.31 ± 0.22 s (*P* < 0.01; recovery 1.30 ± 0.17 s, *P* < 0.05 to control, RM-ANOVA); and Te2 shortened (Fig. 4) from 1.90 ± 0.32 s to 1.13 ± 0.22 s (*P* < 0.01; recovery 1.08 ± 0.18 s, *P* < 0.05 to control, RM-ANOVA). MAP increased from 15.7 ± 0.7 to 18.7 ± 1.3 kPa (recovery 15.5 ± 0.7 kPa, *P* < 0.01 to control and recovery, RM-ANOVA) following bilateral microinjections of KYN. The breathing-related Pkdia, Te1, ETCO2, and heart rate did not change significantly due to the microinjections of KYN.

#### Gabazine.

Microinjection of GAB in the PreBötC-induced pronounced changes in breathing within a few minutes after the microinjection, developing to a maximum within 10–60 min and slowly recovered. After bilateral microinjections, fB decreased (Fig. 4) (63 ± 6% of control, *P* < 0.01 to control and to unilateral microinjections 95 ± 7% and *P* < 0.05 to recovery 101 ± 17%, RM-ANOVA). Ti lengthened (bilateral microinjection 1.99 ± 0.22 s, *P* < 0.001 relative to control 1.09 ± 0.11 s and unilateral injection value 1.11 ± 0.13 s, *P* < 0.01 to recovery 1.29 ± 0.18 s, RM-ANOVA), Te2 increased (bilateral microinjection 2.58 ± 0.37 s, *P* < 0.05 relative to control 1.85 ± 0.42 s, to unilateral injection 1.89 ± 0.29 s and to recovery 1.72 ± 0.23 s, RM-ANOVA) and Te also lengthened (bilateral microinjection 3.03 ± 0.35 s, *P* < 0.05 to control 2.13 ± 0.40 s, to unilateral injection 2.19 ± 0.29 s and to recovery 2.06 ± 0.25 s, RM-ANOVA) (Fig. 4). Decreased MAP was observed (Fig. 4): control, unilateral, bilateral microinjections, and recovery of 13.7 ± 1.3 (*P* < 0.05 to recovery, RM-ANOVA), 11.2 ± 1.5, 10.9 ± 0.7 (both *P* < 0.01 to recovery, RM-ANOVA), and 16.0 ± 1.1 kPa, respectively. GAB microinjections induced no significant changes in Te1, Pkdia, ETCO_2_, and heart rate (RM-ANOVA).

### Phase Timing Correlations

**Figure 5.**
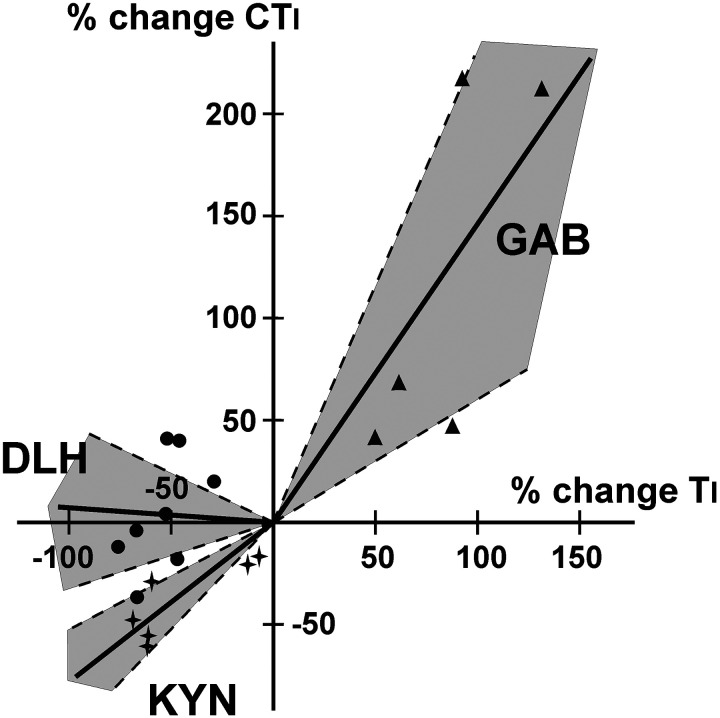
Graph of correlations between changes in inspiratory (Ti) and cough inspiratory (CTi) durations after microinjections of d,l-homocysteic acid (DLH; *n* = 8), kynurenic acid (KYN; *n* = 6), and gabazine (GAB; *n* = 5) in the pre-Bötzinger complex area. Changes are more pronounced for breathing after KYN and for coughing after GAB delivery. Shaded areas encompassed by dashed lines represent 95% confidence intervals.

The percent change in Ti and CTi for DLH microinjections were not correlated (*r*^2^ = 0.06; slope = −0.0827, regression forced through 0; slope n.s. from 0; slope significant from 1, *P* < 0.001). The percent change in Ti and CTi (Fig. 5) from KYN and GAB microinjections in PreBötC were correlated (correlation coefficient *r*^2^ = 0.69 and *r*^2^ = 0.59, respectively). Post KYN microinjection, Ti decreased more than CTi (slope 0.78, regression forced through 0; slope significantly different from 0, *P* < 0.001; slope n.s. from 1; Fig. 5), and post GAB microinjection, CTi increased more than Ti (slope 1.46, regression forced through 0; slope significantly different from 0, *P* < 0.01, slope n.s. from 1). Te and CTe for DLH microinjections were weakly correlated (*r*^2^ = 0.41, slope n.s. from 0). Post KYN and GAB microinjection, changes in Te and CTe were not correlated (*r*^2^ ≤ 0.1).

## DISCUSSION

In this series of experiments, we sought to understand further the role of the PreBötC in the regulation of the cough reflex. Local modification of neurotransmission via glutamate and GABA receptors in the region resulted in substantial changes in the responsiveness and motor pattern of tracheobronchial cough.

The microinjections made in these experiments were in the PreBötC, a critical region of the brainstem that is primarily responsible for the breathing rhythm. Perturbation of neuronal activity in this area was confirmed by extracellular recordings and histological evaluations (Fig. 1).

### DLH

DLH, a synthetic glutamate analog, is an excitatory amino acid agonist. A low dose of DLH was chosen in our experiments to eliminate possible depolarization block of neurons (37). This dose of DLH was still effective in inducing significant changes in breathing and cough. The changes in breathing were relatively short in duration (∼2 min). This limited duration of effect constrained us to unilateral microinjections and focused our analysis on the first two cough bouts postmicroinjection of DLH. Typically, the first cough trial after an extended period of eupnea yields weaker and fewer coughs (18), but we included this trial in the analysis of the effects of DLH to capture the maximal effects of this drug. DLH microinjected into the PreBötC resulted in apneusis and/or rapid breathing with virtually no detectable expiratory duration in cat, rabbit, and rat (1, 8, 28, 32, 33, 40–43). Specifically, in our data set, we also observed apneusis and a rapid breathing pattern, driven by a decrease in Ti and Te and with an increase in inspiratory EMG magnitude.

DLH microinjections induced changes in cough responses distinct from that of breathing. Post DLH, fB and breathing inspiratory EMG activity increased, and Ti decreased markedly, but CN, cough inspiratory EMG activity, and CTi remained virtually unchanged. Statistically significant but mild decreases in CPkps ipsilateral relative to contralateral to the microinjection were observed. This is the first report of perturbation of PreBötC on parasternal motor pathways. Relatively little is known regarding how ipsilateral and contralateral motor drive to the parasternal motor pools are balanced by perturbation of PreBötC neurons. The lack of reduction in CPkdia also confirms previous observations of differential motor control of PS and DIA motor output (44, 45) and extends those results to coughing. Although Te during breathing was substantially shorter following microinjections of DLH, cough-related expiratory timing and CPkabd decreased much less. Based on the differential sensitivity of breathing and coughing to microinjections of DLH, we speculate that this perturbation induced reconfiguration of PreBötC neuronal network during cough. An alternative but not mutually exclusive hypothesis is that specific cough-related neuronal elements in this region underwent selectively altered discharge in response to DLH. One effect of DLH microinjection was enhanced inspiratory tonic activity of neurons in the PreBötC, leading to transient perturbation of the breathing motor pattern. We speculate that one potential mechanism accounting for a relatively normal cough motor drive at this time was a stronger excitatory motor drive during cough. A reduced BP during the postinjection trials could have potentiated cough (46). However, the trajectory of altered BP did not match that of coughing postinjection and the breathing potentiation was much higher than would be induced by the observed decrease in BP. We also cannot completely rule out the disparity in responses of expiratory trunk and abdominal muscles (44, 47) that might be responsible for differing effects on EMG motor outputs and EP recordings. Of note, microinjections of similar low doses of DLH in the more rostral BötC area had little effect on cough inspiratory durations (18).

### Kynurenic Acid

KYN is a nonspecific, broad spectrum glutamate receptor antagonist that induces actions on neurons that are mostly inverse to that for DLH (48, 49). KYN is also an antagonist of glycine receptors and a modulator of GABA_A_ receptors, although direct effects via cholinergic mechanisms [particularly the α7 receptors (50)] have not been confirmed (49). KYN microinjections in the PreBötC induced variable changes in CN that were not statistically significant from control, similar to the effect of DLH microinjection. Even bilateral microinjections of KYN in the restricted area of the PreBötC did not completely eliminate cough motor drive and prevent generation of the cough motor pattern. These findings differ significantly from perturbation of the NTS by this antagonist. Microinjection of KYN into the rostral NTS can completely eliminate coughing and induce a phenomenon analogous to cough apraxia, in which inspiratory and expiratory motor drive increases in a tonic manner during mechanical stimulation of cough receptors with no alternating phasing of the diaphragm and abdominal muscles (23). As such, the evidence thus far provides greater support for nonPreBötC regions, such as the NTS, as the kernel of the cough rhythmogenic mechanism. Nevertheless, cough magnitudes were reduced and a majority of the temporal features of coughing shortened post KYN, suggesting a significant contribution of PreBötC neuronal circuits in the formation of cough motor pattern.

Previous works report various respiratory changes after blockade of excitatory amino-acid neurotransmission or lesioning in the area that were dependent on the exact injection site, the time when breathing was measured, the dosage of drug, and possibly species. Results from these studies included reductions, complete elimination, as well as enhanced breathing (3, 4, 7, 38, 48, 51–53). Our bilateral microinjections of KYN into the PreBötC produced an increase in fB due to shorter Ti and Te. This finding supports the significance of PreBötC neurons and glutamate-related neurotransmission for the generation of respiratory rhythmicity and inspiratory activity.

For cough, we observed changes in phase timing that were similar to those during breathing, such as shorter CTi and CTe following microinjection of KYN. We also observed a shorter period of overlap of the DIA and ABD EMG activity during coughing (the variable over in Table 1), which suggests independent control of inspiratory and expiratory cough-related activity by neurons in the region. Unlike for coughing, Pkdia during breathing did not change after KYN, suggesting significant differences in the contribution of PreBötC mechanisms to inspiratory motor drive for these two behaviors. Furthermore, although Te and CTe were significantly altered in the same direction by KYN and GAB, there was no significant correlation between the magnitudes of change of these phases.

Microinjections of KYN produced long-lasting alterations in phase timing and very different effects on Pkdia and CPkdia. The magnitude and temporal features of cough are regulated in an independent manner (9), and our results regarding the recovery of spatial and temporal control of coughing following microinjections of KYN support this concept. Although the magnitude of cough inspiratory drive and inspiratory phase timing of cough exhibited strong recovery after microinjection of KYN, the inspiratory phase duration of breathing did not recover in a time span of up to 3 h. Microinjections of KYN apparently induce differing long-term effects on the control of inspiratory phase duration for these two behaviors by unknown mechanisms. This finding supports the existence of selective control of cough inspiratory duration relative to that for breathing by glutamate-related mechanisms in, or functionally interacting with, the PreBötC.

We believe that changes in cardiovascular output did not contribute to the decrease in cough EMG magnitude, as CN did not change in response to the increase in BP (46). In addition, although BP recovered with time, cough EMG magnitude did not. We also observed no change in heart rate postinjection. Dillon et al. (51) reported an increase in heart rate due to KYN injections into the rostral ventrolateral medulla, but we microinjected caudal to the injection sites reported in that study.

### Gabazine

The PreBötC, which is crucial for inspiratory rhythmogenesis, critically involves interactions of inhibitory neuronal circuits (2, 35). Our unilateral and bilateral microinjections of GAB, a GABA_A_ receptor antagonist, into the PreBötC decreased fB due to a prolongation of both phases of breathing, which is consistent with previous literature in several vagotomized species (2, 5, 54). Significant impairment of GABA-related inhibition in the region may result in the dysfunction of cycling properties of the network and consequently produces suppression of spatial and temporal components of breathing. GABA-ergic inspiratory neuronal populations within the PreBötC provide phasic inspiratory inhibition to many parts of the brainstem (55, 56) and participate in shaping the inspiratory pattern and coordinating inspiratory and expiratory activity (55, 57).

Microinjections of GAB into the PreBötC decreased CN and expiratory cough strength after both unilateral and bilateral injections, and these changes slowly (within 2–3 h) recovered. Similar to breathing, phase durations of the cough motor pattern were prolonged including the decrementing part of the cough inspiratory activity (Table 1). The GAB-induced depression in synaptic inhibition resulted in a delayed phase transition during coughing. This interesting result may correspond to a significant involvement of a GABA_A_ receptor-mediated mechanism in inspiratory-expiratory phase transition during breathing (21). However, neither breathing nor coughing inspiratory magnitudes (EMGs as well as esophageal pressure) were altered in a statistically significant manner aside from an increased variability during cough. These findings are consistent with a significant role of GABA_A_-dependent inhibition in phase transition and phase duration control during coughing with a dominant role of neuronal excitation in control of inspiratory motor drive from the PreBötC for both breathing and coughing.

The strong relationship of GABA-ergic inhibition in the PreBötC with the control of cough magnitude, particularly the decrease in cough expiratory efforts, is important. All data on the PreBötC indicate its importance for the production of inspiratory motor patterns (including cough, see KYN data). Thus, it was surprising that cough expiratory suppression was induced by disruption of GABA_A_-related inhibition in the PreBötC. Expiratory abdominal motor drive during coughing is ballistic-like and can be as much as an order of magnitude larger than even maximal breathing-related activation (58), indicating the requirement of significant brainstem excitatory input to expiratory premotor neurons. One explanation for this effect could be that PreBötC circuits directly or indirectly excite neurons that participate in cough-related expiratory pathways.

### Limitations

Vagal afferents have a role in modifying both eupnea and cough. Because our experimental model is unparalyzed, spontaneously breathing and vagal-intact, these vagal afferents, such as volume-related feedback, may confound a direct comparison of effects between eupnea and cough.

DLH microinjections were limited to unilateral administration. As we noted in the methods, the duration of action of DLH is very short, and that limited our ability to microinject this drug on the contralateral side within the timeframe of the effect of the initial injection.

Bilateral vehicle control (aCSF) microinjections into the PreBötC decreased CN significantly. One explanation for the decrease in CN and CN% is due to volume displacement from the microinjection. However, the change in CN due to bilateral injection of aCSF in the PreBötC is small and not physiologically significant compared with the change in CN due to DLH, KYN, or GAB microinjections.

### Conclusions

In conclusion, our results indicate that *1*) the PreBötC is important for the generation of the cough motor pattern; *2*) excitatory glutamatergic neurotransmission in the PreBötC does not play an important role in the cough rhythm generation; however, it is involved in control of cough intensity and patterning; *3*) GABA_A_ receptor-related inhibition in the PreBötC strongly affects phase durations of breathing and coughing in the same manner, suggesting involvement of common neuronal populations in control of temporal characteristics for these motor acts; *4*) surprisingly, cough expiratory efforts markedly depend on inhibition via GABA_A_ receptors in the PreBötC; and *5*) perturbation of breathing magnitude and phase timing does not obligate similar changes in cough strength and phase timing, supporting the potential for separate control of these behaviors by mechanisms involving the PreBötC.

The findings are consistent with the regulation of cough by a highly dynamic distributed and cooperative network of neurons, that spans several important control sites in the brainstem, and that may reconfigure assemblies and change firing rate range depending on the produced behavior (1, 9, 10, 23, 26, 35, 59–61).

## GRANTS

The study was supported by NIH R01HL131716, NIH SPARC 3OT2023854, and VEGA 1/0092/20.

## DISCLOSURES

No conflicts of interest, financial or otherwise, are declared by the authors.

## AUTHOR CONTRIBUTIONS

T.Y.S, I.P., T.P., and D.C.B. conceived and designed research; T.Y.S., I.P., M.J.R., M.N.M., Z.K., and L.M. performed experiments; T.Y.S., I.P., and L.M. analyzed data; T.Y.S, I.P., and D.C.B. interpreted results of experiments; I.P. prepared figures; T.Y.S. and I.P. drafted manuscript; T.Y.S., I.P., T.P., P.W.D., and D.C.B. edited and revised manuscript; T.Y.S., I.P., M.J.R., M.N.M., Z.K., L.M., T.P., P.W.D., and D.C.B. approved final version of manuscript.
